# Small business success: Identifying safety hazards and safety risks

**DOI:** 10.4102/jamba.v11i1.767

**Published:** 2019-08-19

**Authors:** Elriza Esterhuyzen, Leonie B. Louw

**Affiliations:** 1 Department of Operations Management, University of South Africa, Pretoria, South Africa

**Keywords:** safety hazards, safety risks, safety management, small business management, entrepreneurship, risk management

## Abstract

The establishment and growth of the small businesses in South Africa is of vital importance for economic stability and progress in the economy. A key small business management skill comprises occupational health and safety management, with particular reference to the proper identification of safety hazards and safety risks. This conceptual article set out to identify current perceptions about the concepts of safety, safety risks and safety hazards in the workplace while also identifying and analysing misconceptions regarding safety hazards. This article reports on a secondary data analysis of relevant literature on safety hazards and safety risk and the role thereof on small business success. The structure and functioning of the interrelated characteristics of safety hazards were visualised, with the objective of allowing small business owners or managers to understand how safety hazards contribute to safety risks. Proper identification of safety hazards and safety risks, along with adequate protection measures, allows for improved productivity and a reduction in operational costs. Safety hazards in the workplace, encompassing the functional and structural characteristics, such as humans and machinery, should be noted by small business owners, as applicable to all types of businesses. This article may serve as a catalyst for small business success through growth and sustainability by implementing enhanced safety management practices based on an accurate identification and analysis of safety hazards and safety risks. It allows for the identification and proactive mitigation of safety hazards and safety risks in all types of small businesses.

## Introduction

South Africa is currently experiencing major challenges pertaining to economic sustainability and economic progress. The establishment and growth of small businesses in South Africa has been identified as a potentially significant contributor to address such challenges (DTI 2014:11). Small businesses are seen as pivotal to the growth and development of the South African economy, with safety management being indicated as one of the skills required to ensure the sustainability of such businesses (Naidoo 2010). According to Okoye and Okolie (2014:21), the successful management and financial growth of small businesses depend on the proper implementation of occupational health and safety policies and procedures, which is based on the proper identification of safety hazards and safety risks.

Traditionally, the fundamental issue concerning safety is related to the threat or risk posed by certain factors or aspects to the well-being (safety) of humans, as well as to the condition of the natural and human-created environment. If there were no threat to the safety (safety risk) of the people or immediate natural and human-created environment (non-natural, developed), the concept of safety would not exist because it would not apply and would be irrelevant. In the small business environment, accidents can cause injuries or death, environmental damage and can ultimately damage the reputation of the business, all leading to reduced profitability (Okanga & Groenewald 2017).

The study of workplace safety starts by examining the origin(s) and existence of the threats (safety risks) that some factors or aspects present to the safety of humans, as well as the natural and the human-developed (non-natural) environments. Such an examination also needs to generate answers about the nature of the factors or aspects that pose a safety risk. In addition, it could contribute to the current knowledge regarding workplace safety, as well as specifying the overall context of safety in relation to other frameworks of knowledge and objects of study. Small business owners or managers need to have an understanding of workplace safety to enable them to properly identify relevant safety hazards and safety risks in their respective small businesses. Employee well-being and improved working conditions should be of particular importance to small business owners or managers as part of enhancing profitability as well as employee morale. Okanga and Groenewald (2017) posit that the proper identification of safety hazards and safety risks allows for the proactive minimisation of health and safety risks for both employees and the community.

### Ethical considerations

Ethical clearance for this conceptual article was obtained from the University of South Africa, Department of Operations Management Ethics Review Committee with reference: OM/2018/004.

## Perceptions about safety as the object of study

Small business owners or managers need to be able to qualify the object of study regarding safety in the workplace. In this regard, Bonacea (n.d.:1) indicates that the term ‘object’ relates to the phenomenon that the research subjects to cognitive attention and observations. The study object is fundamental to all investigative processes that are involved in perceiving and analysing an element of reality. The object of study represents an observable ‘something’ of material that is perceivable by human senses. Therefore, safety as the study object needs to be clearly defined. Such a definition must stipulate the applicable limits, and clarify any uncertainties there might be among the group of humans that research the identified ‘something’. The small business owner or manager, who studies the observable phenomenon of safety in the small business, needs to clearly identify, understand and verify the object of study, which can and must be independent of the cognition and reasoning of the small business owner or manager. The object of study must be observable, generalised and independent from the cognition and reasoning of the small business owner or manager, and it must apply to a total universal context. The study object may be physically concrete or abstract. These requirements apply to safety.

A good example of an abstract study object can be found in the science of sociology. Sociology lists its study object or object of study as all social aspects of the community in its total context (Carlson 2017:1). The focus is on all the elements of human relationships that fall under the term ‘community’. The social relational aspect of community is not a concrete element of society, but it is observable, well qualified, although limitedly, and applies universally and in the total context. Safety may link intensely to this approach as will later be qualified. The basic departure for establishing safety as the object of a study is to identify the fundamental point of focus, namely, the origin of safety risk.

## The origin(s) of safety risk

Safety risk is the most important element of safety. Questions that apply in this respect are: Where and when does safety risk start? What ‘things’ can pose threats or risks to the safety of people and the environment, and when? The following three issues are relevant in this context:

- Firstly, in a real sense, only things with which contact can be made and which are tangible can pose a safety risk. Such things are made of matter, the universal substance that occupies space and has mass (Zumdahl & Zumdahl 2007:25). If something cannot contact anything else, no threat or no safety risk can be created or originated. Therefore, no contact, no threat, and no threat, no safety risk. For something to create a safety threat, that object must possess contact or collision potential (Van Fleet 2000:112).
- Secondly, for something to create a threat to safety, that thing must make contact with something else, and some interaction must occur between the objects when they collide. If there is no contact or no interaction, no safety risk can be created.
- Lastly, the adverse effects of the threats will depend on the level of safety risk resulting from the intensity of the interaction and the energy exchange occurring between the things or substances that are involved in the process.

### The creation of threats to safety

Only something that is able to make contact with something else can create a threat to safety. To be contactable or make contact requires that objects be made of matter. If an object is not made of matter, in effect, it means that it is non-contactable; therefore, it cannot contact other things and has no collision potential. So, something that does not have a matter base cannot create a safety threat. Matter provides the substance for the contactability between things. Therefore, things that are contactable are known as substances and, because substances are contactable and can create safety risk, all substances can be regarded as safety hazards.

### The process of creating safety risk

Whenever substances (safety hazards) make contact with one another, they become involved in some happening(s) involving both of them. Such happenings can be regarded as a form of interaction that occurs between the things that are in contact with each other.

The happenings or interactions between substances result in the origination of circumstances that create one or more threats to the safety of some or all of the substances that are involved in the interaction or that are found in the immediate environment. Substances in the immediate environment may be affected by the outcome of the interaction. Under normal circumstances, if substances do not interact, no threat is created to threaten the safety of the substances involved or any other substance in the immediate vicinity. The threats that are created and that threaten the safety of the substances are called safety risks.

Thus, a safety risk is created or originates as result of the contact and interaction between ‘things’ or substances (safety hazards). During the interaction of the substances, some form of energy exchange occurs. Safety threats or safety risks originate from the energy exchange during the interaction of two or more substances. However, safety risk can also be created if substances are expected or supposed to make contact and to interact, but refrain from doing so. Thus, if substances do not make contact and do not interact as required, expected or supposed to, a safety risk may also be created. This implies that the lack of energy exchange could also contribute to the generation of safety risk.

### Unambiguousness of substances that create safety risk

If something (substance) does not have a matter base, it cannot contact or be involved in some happening with something else (substances). Substances with a matter base never lose their ability to make contact and to interact with other substances with a matter base. They remain unambiguously true to their tangibility and interactability. The laws of nature apply to substances with a matter base, and symmetrically force them to unambiguously adhere to their abilities and characteristics. Later in this article, more will be explained about the characteristics of substances as well as natural laws and symmetry.

One needs to understand and realise that tangible substances unambiguously remain substances and that they have the potential to make contact and to interact with other substances, irrespective of circumstances. Substances keep their contactability and interactability with other substances, irrespective of changes in their nature. Substances (things) unambiguously remain creators of safety risk as result of their irreplaceable contactability and interactability.

### Safety hazards and safety risk

Substances create safety risks as a result of making contact with and their interaction with other substances. In this context, substances are safety hazards. The concept of safety hazards is not new, with Grimaldi and Simonds (1989:181) having defined a safety hazard as ‘the source of energy … which, when uncontrolled, leads to harmful occurrences’. Safety hazards are part of everyday life, also in small businesses, where safety risks are generated through the contact and interaction with other safety hazards.

Stephenson (1991:8) contends that something that can cause harm is a safety hazard. The safety risk originates from the contact and interaction of safety hazards, which can complement human activities or interfere with human activities (Van Fleet 2000:112). Drinking pure water versus drinking toxic water serves as a good example in this regard. However, the opposite is also true. Safety risk can also result when safety hazards do not make contact or do not interact as they are supposed or expected to do under given circumstances. The safety risk that results from such lack of contact, lack of interaction or ineffective interaction can also complement or interfere with human activities. For example, when a patient needs to inhale oxygen but the medical cylinder is not turned on, no contact or interaction between the safety hazards is possible that creates unnecessary safety risk to the patient.

All substances have a matter or material basis. Thus, all substances are safety hazards. All safety hazards offer a safety risk; therefore, there are no circumstances on earth or in life that do not offer or experience safety risk. Humans need safety hazards to live and enrich their lives, therefore humans continuously engage or interact with safety hazards every day. This also applies to the business environment where constant interaction between humans and safety hazards is required in terms of manufacturing, retail and services, which supply their needs. For example, within a manufacturing environment, the raw material, equipment and humans are required to produce goods. However, these same raw materials, equipment, humans and goods are safety hazards in themselves, as per the definition below. The same applies to the retail sector, where interaction between humans, and between goods and humans, is inherently required as part of business activities. Service delivery requires the same interaction between various safety hazards, such as human interaction and interaction between humans and tools of the trade. Safety hazards are also responsible for adverse effects that result from the engagement or interaction between the safety hazards, which, in numerous instances, also involve humans. Safety hazards and safety risk are indispensable elements of the daily life of humans.

## Definition of safety hazard(s)

Considering the preceding reasoning, it requires that the term ‘safety hazard’ be clarified to enable an understanding of the context of the origin of safety risks via tangible, interacting and unchangeable or unambiguous substances, which are safety hazards. The definition of safety hazards distinguishes between real safety hazards, unacceptable hazards and potential safety hazards.

### Real safety hazards

A safety hazard is something that

- is a substance with a matter base that can make contact (collide) with other substances
- can interact with other substances by being involved in some activity or happening that can result in the creation of one or more threats or risk to the safety of other substances
- remain unambiguously true or unambiguously adheres to its ability to make contact and interact with other substances.

By implication, this definition confirms that all tangible substances or objects that have a matter base are safety hazards. This applies to all substances, without exemption.

### Unacceptable safety hazards (misconception)

The reasoning thus far adamantly proves that ‘anything’ or ‘something’ that does not have a matter base cannot contact or cannot interact with other substances. The reasoning furthermore confirms that substances that do possess these two capacities are safety hazards. By the same reasoning, therefore, anything that does not have the two capacities cannot be a safety hazard.

The literature on safety (Goetsch 2010:5; Hansen 2012:68; Sacks et al. 2015:60; Smith 2012:370) has listed several (at least 10) definitions of ‘things’ that are postulated to be safety hazards that can cause safety risk (Smit & Esterhuyzen 2014:10–16). These ‘things’ that authors have listed and explained are not contactable because they do not have a matter base. As result of their intangibility, they cannot make contact (collide) or interact with other tangible things. In addition, the definitions as listed in the literature do not have the capacity to remain safety hazards unambiguously always and in all circumstances. The lack of these three abilities implies that all such ‘things’ cannot be involved in creating safety risks. Therefore, all such things that are not tangible have no interactability and that are not consistently unambiguous can never be safety hazards (Smit & Esterhuyzen 2014:23–29). An example in this regard is a ‘situation’ that many authors (Goetsch 2005:294, 577; Hansen 2012:68; Sacks et al. 2015:60) refer to as a safety hazard. Is a situation contactable? Is it made of matter? Can a situation interact with other situations? Or is a situation, in fact, only the creation of the human mind?

Table 1 confirms the existence of definitions that authors define as safety hazards, but which do not meet the basic requirements of being real safety hazards in terms of the three abilities discussed.

**TABLE 1:** Contextual assessment of definitions of a safety hazard.

Current definitions of safety hazards in the literature	Criteria for a real safety hazard						Real hazard?	
	Contactability		Interactability		Unambiguousness			
	Yes	No	Yes	No	Yes	No	Yes	No
A situation is a safety hazard	-	X	-	X	-	X	-	X
A condition is a safety hazard	-	X	-	X	-	X	-	X
A predisposition is a safety hazard	-	X	-	X	-	X	-	X
A method and process or work practice is a safety hazard	-	X	-	X	-	X	-	X
A human act is a safety hazard	-	X	-	X	-	X	-	X
Exposure is a safety hazard	-	X	-	X	-	X	-	X
Departing from normal is a safety hazard	-	X	-	X	-	X	-	X
A source of physiological and behavioural factors that causes harm or damage is a safety hazard	-	X	-	X	-	X	-	X
Safety risk associated with loss or damage is a safety hazard	-	X	-	X	-	X	-	X
Stress is a safety hazard	-	X	-	X	-	X	-	X
An event is a safety hazard	-	X	-	X	-	X	-	X

*Source:* Smit, S.J. & Esterhuyzen, E., 2014, *The basics of safety hazards and the origins of safety risk*, Business Print, Pretoria. p.30.

The items identified in the current definitions of safety hazards do not have any of the listed characteristics of real safety hazards. Thus, they are not, and can never be, safety hazards.

### Potential safety hazards

The reasoning and argumentation thus far postulate that all substances that have a matter or material base, are tangible and can interact with other substances while unambiguously remaining constant are safety hazards. This implies that if a ‘thing’ or substance is a safety hazard, it stays a safety hazard under all circumstances. Such a safety hazard cannot be a safety hazard and a potential safety hazard. To be a potential safety hazard means that a substance is not a safety hazard under specific circumstances, but can become or will develop into a safety hazard under similar or different circumstances. It is imperative to recognise that such an assumption is totally invalid about all substances. All substances exist as safety hazards because of their material base. None of the ‘so-called safety hazards’ listed in Table 1 has the potential to develop or become a safety hazard, ever.

So, the idea and definition of a potential safety hazard is invalid and unacceptable because once a safety hazard, always a safety hazard. Potential safety hazards do not exist at all.

### Characteristics of safety hazards

All safety hazards have the same types of characteristics that relate to their material base. The characteristics of safety hazards provide the perspective on understanding the structure and functioning of specific safety hazards. Every safety hazard possesses nine different interrelated characteristics, of which six relate to its *structure* and four to its *function* (Smit & Esterhuyzen 2014:45–48), as will be discussed in the below section.

#### The structural characteristics of safety hazards

The structural characteristics of safety hazards pertain to their structural features. Such structural characteristics are the following:

- *Tangibility* that relates to the material base, which causes all substances to be physically contactable.
- *Density* that relates to the amount and compressed nature of the matter and that sets the basis of solids, liquids and gases.
- *Size* that relates to the volume (amount) and spread (distribution) of the material base.
- *Weight* that relates to the gravitational pull of the material base towards the centre of the earth.
- *Shape* that relates to contour of the spread of the material base.
- *Texture or surface* that relates to the evenness of the spread of the matter base on the peripheral or outer contour of the material base.

#### The functioning characteristics of safety hazards

**FIGURE 1:**
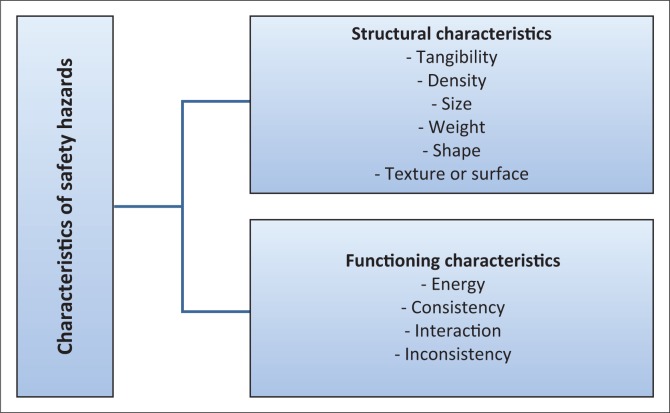
Characteristics of safety hazards.

All safety hazards display a common way of functioning (Smit & Esterhuyzen 2014:58–59). Issues in this regard are the following:

- *Energy* that relates to the kinetic energy, which operates within the atoms of the safety hazards that are tangible substances. The term ‘energy’ implies the capacity to do work (Kramer 2015:4). In this case, safety hazards have functional energy. Every safety hazard possesses two basic energies, namely, kinetic and potential energy. Kinetic energy implies the capability to move, while potential energy implies the capability to operate in different types of energies that can be brought onto the safety hazard. The concept of ‘different energies energy’ can be defined as the potential or configuration of energies (Kramer 2015:6).
- *Consistency* relates to the capability of safety hazards to operate in symmetrical ways that are predetermined by natural laws. Safety hazards operate in unique and consistent ways that generally cannot be changed.
- *Interaction* relates to the capability to exercise influence on other safety hazards. When safety hazards make contact, they go into an energy-exchanging mode with each another. This exchange of energies results in different effects on different safety hazards involved in the process of interaction. There are different modes of safety hazard interaction (Smit & Esterhuyzen 2014:85).
- *Inconsistency* relates to the human tendency towards inconsistent behaviour. Therefore, the human is classified as a safety hazard and the preceding list of characteristics of safety hazards fully apply to the human being. Although the body and organs of the human function on symmetrical consistent bases, the mind functions on an uncontrollable and inconsistent basis. Inconsistency is also common in animals. However, while the inconsistency of the human results from the rational thinking process of the human mind, the inconsistency of animals occurs because of instinct. Animal instinct and behaviour are relatively controllable under most circumstances, although they could choose to follow their instincts. By contrast, humans may, can or will do as they deem fit (Figure 1).

## Influence of safety hazard characteristics on safety risk

The characteristics of safety hazards contribute to the creation of safety risk in a situation. For example, the characteristics can also contribute to the creation of the level(s) of safety risk in terms of high or low safety risk within a situation. While fuel in the form of vapour can contribute to the safety risk of an explosion, the extent of the explosion will differ based on the volume (size) of the vapour.

However, the contribution of the characteristics of the safety hazard risk is not directly related to the direct opposite of some of the characteristics, for example, big versus small, heavy versus light, sharp versus blunt, hot versus cold and so forth. The contribution of safety hazards to the creation of safety risk is always directly related to the specific circumstances found in a situation. No two situations have similar safety risks in terms of nature and level.

### Definition of safety risk

The concept of ‘safety risk’ represents a measure of uncertainty or adversity regarding safety (Fuller & Vassie 2004:5). Such uncertainty refers to the outcome of issues that people value (Fischhoff & Kadvary 2011:22). The outcome relates to the results of the actual or lack of contact and interaction between safety hazards (Smit & Esterhuyzen 2014:142). Safety risk results from the actual lack of contact and interaction of safety hazards. Safety risk represents the existence of threats to the well-being of humans, as well as to the man-made or developed and natural environment.

### Definition of safety

Safety represents the acceptable freedom from threats to the well-being of humans, as well as the natural and developed surrounding environment. Such freedom is always relative because safety always depends on the competence of the humans and the threshold limits of the man-made and natural environment to withstand the possible or potential adverse effects of the levels of safety risk present in the situation.

Safety also represents the study activities that develop a specific frame of knowledge pertaining to enhancing safety, also in the workplace. Hollnagel (2013:1) postulated that ‘safety science is the study of safety’, but because safety has no matter basis, Hollnagel questioned his own statement. However, considering that safety risk originates from safety hazard interaction, such questioning does not apply. It is at this point where the issue of safety as the study object becomes relevant.

### Safety as the study object

Science presents a system of research and knowledge. Researchers use models to describe a process and the results of a study. These models serve the purpose to make postulations that can be validated through observation and experimentation. Scientific theory provides logical models or frameworks that examine the behaviour of different phenomena. A phenomenon represents an ‘object of a person’s perception or the sense that the mind notices’ (Reader’s Digest Oxford Complete Word Finder 1993), and, in philosophy, the ‘object of a person’s perception’ is that which a person can perceive (Kavanagh & Pearsall 2006). Scientific method seeks to explain the complexities (difficulties) of nature (life) in a common, known and easily replicated way, and to use the explanations to make useful predictions about any aspect of reality, for example, flora, nature, economics, capital, zoology, arts and numerous more (Sheikh 2009:6).

Scientific method enables a study to become a reality by focusing on a specific study object. Such a study object comprises a single phenomenon that serves as the final object of the study. The object of study of scientific research is without exception a specific object, within a specified relation (Sheikh 2009:7). Different sciences conduct studies of different phenomena, for example, the human body, the nature of human behaviour, the healing of the human body, human relations, the way people learn and many more.

A system of study has a specific study object. Safety focuses on studying the ways in which things make contact and interact (interface) in different situations, as well as the results of such interactions (interface). The primary focus is on the man–machine interaction or interface within a given environment, such as a small business. Safety hazards in the workplace, encompassing the functional and structural characteristics, such as humans and machinery, should be noted by small business owners, as applicable to all types of businesses.

‘Man’ refers to human, while ‘interface’ refers to any means of interaction, and ‘machinery’ implies ‘any article or combination of articles assembled, arranged or connected and which is used or intended to be used for converting any form of energy to performing work, or which is used or intended to be used, whether incidental thereto or not, for developing, receiving, storing, containing, confining, transforming, transmitting, transferring or controlling any form of energy’ (South African Government 1993:413). This focus on safety related to the man–machine interface considers design, displays, communication, automation, contact, interaction and many more (Stranks 2006:136–137). The focus of safety is on the interaction of safety hazards, the results in the form of safety risks (threats) and the effects of such threats on the surrounding environment.

The field of safety studies the actual and lack of interaction, and the outcome and effects of safety hazards. Such a focus is on the unexpected and adverse results of safety hazard interaction. Based on the study of the successful or effective interaction of safety hazards that include humans and the resultant safety risk level(s), safety describes and sets proactive preventative guidelines or models for safe interaction. On the contrary, as a result of the study of the unsuccessful or ineffective interaction of safety hazards, which include humans and the resultant safety risk level(s), safety describes and sets re-active preventative guidelines (models) for successful interaction or interface. To set guidelines for safe interaction, it is important that the managers of small businesses understand the nature of all safety hazards that are involved in the interaction in each situation. The vast extent of safety hazards that are involved requires that safety works in close cooperation with multi-disciplinary cross-functional teams with the view to understanding the nature and outcome or effects of safety hazard interactions.

## Conclusion

To be sustainable and socially responsible, it is vital that small business owners or managers accurately evaluate the safety hazards and safety risks in their business, and take the necessary steps to mitigate or avoid these safety hazards and safety risks as far as reasonably practicable. The safety management skills of small business owners or managers have been identified as being of vital importance in successfully managing a small business (Louw 2018). A small business cannot focus on profitability alone, and the interests of all the stakeholders of the business, including employees, should be considered (Ofori, Nyuur & S-Darko 2014). By minimising the safety hazards and risks, the business will experience improved productivity and a reduction in the operational costs of the business. However, adequate protection measures cannot be put in place if the relevant safety hazards and safety risks have not been properly identified (Okanga & Groenewald 2017). Even though South African business owners or managers do not always regard employee assistance programmes, including safety training, as an important part of business activities, it can potentially have a great impact on business processes and the interaction thereof with individuals’ output and well-being (Matlhape 2003).

In conclusion, it can be confirmed that the field of safety studies the interaction of safety hazards and the effects thereof in different circumstances in real life, such as in small businesses. The study object of safety can be constituted as the interaction of safety hazards and the effects of the resultant generation of safety risks. Such an object of study can be qualified as an integrated part of the material reality that can be perceived by the human senses. The study object of safety is a phenomenon vested as an observable practicality.
